# The *Drosophila* Gene *CheB42a* Is a Novel Modifier of Deg/ENaC Channel Function

**DOI:** 10.1371/journal.pone.0009395

**Published:** 2010-02-24

**Authors:** Yehuda Ben-Shahar, Beika Lu, Daniel M. Collier, Peter M. Snyder, Mikael Schnizler, Michael J. Welsh

**Affiliations:** 1 Howard Hughes Medical Institute, Chevy Chase, Maryland, United States of America; 2 Department of Internal Medicine, Roy J. and Lucille A. Carver College of Medicine, University of Iowa, Iowa City, Iowa, United States of America; 3 Department of Molecular Physiology and Biophysics, Roy J. and Lucille A. Carver College of Medicine, University of Iowa, Iowa City, Iowa, United States of America; 4 Department of Biology, Washington University in St. Louis, St. Louis, Missouri, United States of America; Harvard University, United States of America

## Abstract

Degenerin/epithelial Na^+^ channels (DEG/ENaC) represent a diverse family of voltage-insensitive cation channels whose functions include Na^+^ transport across epithelia, mechanosensation, nociception, salt sensing, modification of neurotransmission, and detecting the neurotransmitter FMRFamide. We previously showed that the *Drosophila melanogaster* Deg/ENaC gene *lounge lizard* (*llz*) is co-transcribed in an operon-like locus with another gene of unknown function, *CheB42a*. Because operons often encode proteins in the same biochemical or physiological pathway, we hypothesized that CHEB42A and LLZ might function together. Consistent with this hypothesis, we found both genes expressed in cells previously implicated in sensory functions during male courtship. Furthermore, when coexpressed, LLZ coprecipitated with CHEB42A, suggesting that the two proteins form a complex. Although LLZ expressed either alone or with CHEB42A did not generate ion channel currents, CHEB42A increased current amplitude of another DEG/ENaC protein whose ligand (protons) is known, acid-sensing ion channel 1a (ASIC1a). We also found that CHEB42A was cleaved to generate a secreted protein, suggesting that CHEB42A may play an important role in the extracellular space. These data suggest that CHEB42A is a modulatory subunit for sensory-related Deg/ENaC signaling. These results are consistent with operon-like transcription of *CheB42a* and *llz* and explain the similar contributions of these genes to courtship behavior.

## Introduction

Organization of genes in operons provides a mechanism for coordinating the quantitative, temporal, and spatial transcription of genes that contribute to the same biochemical or physiological process [1], [2]. Although operons are abundant in archea and bacteria, they are thought to be rare in eukaryotes outside the nematode lineage [1], [3]. We recently reported the existence of operon-like loci in *Drosophila* [4]. The first operon-like locus we identified included a degenerin/epithelial Na^+^ channel (DEG/ENaC) gene (*lounge lizard*; *llz*) co-transcribed with *CheB42a*, a gene with unknown function [5], which resides less than 100 bp upstream [6]. Previous work suggested that *llz* (also called *ppk25* [7]) contributes to male courtship behavior because mutations in the gene result in delayed male courtship [7]. The contributions of *CheB42a* to male courtship have been less clear. *CheB42a* mutations were reported not to alter male courtship [7], but a later publication suggested that *CheB42a* increased male courtship behavior [8]. Furthermore, it was suggested that *CheB42a* expressing cells might enwrap gustatory neurons that express the gustatory receptor *Gr68a*, a gene that has also been identified as essential for courtship behavior [8], [9]. Organization of *CheB42a* and *llz* in an operon-like structure and the contribution of both genes to courtship behavior (although in somewhat opposing ways) suggested that the protein products of *llz* and *CheB42a* might functionally interact.

*llz* is a member of the DEG/ENaC family of genes that encode non-voltage gated cation channels [10], [11], [12]. Like other DEG/ENaC proteins, LLZ is predicted to contain two transmembrane domains, intracellular N- and C-termini, and a large extracellular domain with fourteen conserved cysteines and several small conserved amino acid motifs. DEG/ENaC channel subunits combine to form homo-and/or hetero-multimeric channels comprised of three subunits [13]. Some DEG/ENaC channels are regulated by interactions with ligands; for example, extracellular protons gate acid-sensing ion channels (ASICs) [14], and the peptide FMRFamide gates the *Helix aspersa* FaNaCh [15]. DEG/ENaC genes also contribute to salt absorption [16], mechanosensation [17], nociception [18], and learning and memory [14]. Although all DEG/ENaC proteins are thought to form ion channels, in many cases their ion channel function has not been demonstrated, presumably because we do not know the appropriate ligand or regulatory mechanism.

**Figure 1:**
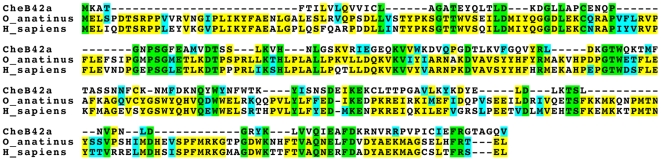
*CheB42a* encodes a protein with sequence similarity to aryl sulfotransferase 1A. Top line shows predicted aminoa acid sequence of *Drosophila melanogaster CheB42a*, and second and third lines show predicted amino acid sequences of *Ornithorhynchus anatinus* (platypus) and *Homo sapiens* (human) aryl sulfotransferase. Green, conserved residues; yellow, conserved residues in two of the aligned proteins; blue, similar residues. Accession numbers for the aligned proteins are: CheB42a, NM_206043.2; O_anatinus, NP_001121091; H_sapiens, NP_003157.1.

*CheB42a* is a member of a family of at least 12 *Drosophila* genes of unknown molecular function. Members of this family are predicted to have a single transmembrane domain, a short intracellular N-terminus, and a larger extracellular domain. The CHEB42A extracellular domain shows limited sequence similarity to aryl sulfotransferases of the SULT1A subfamily (Fig. 1). Recent work speculated that *CheB* genes are homologous to the mammalian Tay-Sachs Gm2-activator protein [5], a lysosomal co-factor involved in the degradation of the ganglioside GM2 [19].

The predicted protein structure of CHEB42A also indicated it might interact with LLZ. First, the CHEB42A structure resembles that of the accessory subunits of other ion channels: an example is the human protein MiRP1, which associates with the HERG K^+^ channel [20]. In addition, the *C. elegans* protein MEC-6 has a similar general predicted structure to the CHEB42A protein and associates with the DEG/ENaC channel subunits MEC-4 and MEC-10 [21]. Accessory subunits can alter the gating and regulation of ion channels [22] and/or serve as a chaperone to regulate the level of channel presence on the cell surface [23]. Thus, we considered that CHEB42A might associate with LLZ and modulate its function. Second, the predicted CHEB42A structure is similar to that of some odorant binding proteins in which a transmembrane segment can act as a signal peptide, anchoring an extracellular odorant binding domain that is released from the membrane following protease cleavage [24]. Thus, we considered that CHEB42A might be proteolytically released from the membrane and interact with LLZ as a secreted protein.

Based on this background, we hypothesized that CHEB42A and LLZ are functionally related and that CHEB42A might act by directly modulating LLZ channel functions in a cell-autonomous fashion. Such a relationship might explain their contribution to similar behavioral processes.

## Results

### *CheB42a* and *llz* Are Expressed in Male Chemosensory Structures

Our earlier *in situ* hybridization in embryos revealed exactly the same pattern for *CheB42a* and *llz* expression; the heads of late stage larvae showed these genes expressed in two classes of sensory neurons: gustatory-like external sensory neurons and putative mechanosensitive multidendritic neurons [4]. These results suggested that the same cells express both *CheB42a* and *llz* [4]. Studies of adults suggested that the locus is expressed in cells associated with external chemosensory bristles on male legs, suggesting that these genes play a role in chemosensation [6], [25]. However, the close contact between males and females during the courtship ritual suggests that mechanosensation might be involved as well [26].

**Figure 2:**
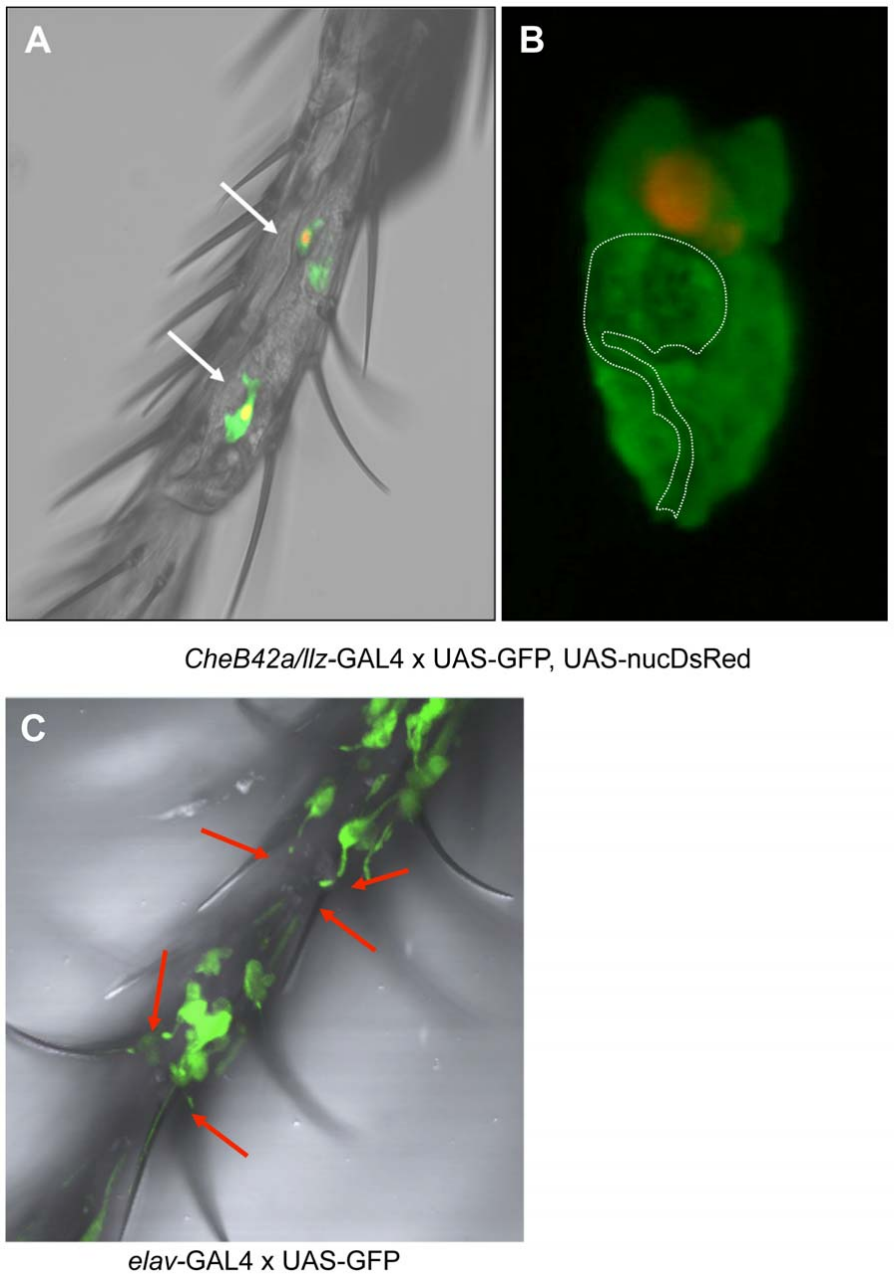
The *CheB42a/llz* locus is preferentially expressed in non-neuronal sensory structures. **A–B.** Driving expression of GFP with the *CheB42a*/*llz* promoter revealed expression in cells in the front legs of males. Co-expression of GFP and DsRed containing a nuclear localization signal revealed expression in relatively large cells with a single labeled nucleus. Morphology of the *CheB42a*/*llz*-expressing cells suggested the cells might enwrap another cell type; white dotted line represents boundaries of a potentially enwrapped cell with neuronal morphology (see Movies S1 and S2 for 3-D reconstruction of typical *CheB42a*/*llz* cells). **C.** Pan-neuronal *elav* promoter driving GFP expression in the same male leg segment as shown in panel A. Typical neuronal sensory morphologies such as sensory cilia extending to the sensory hairs are apparent.

To identify specific cells expressing the *CheB42a*/*llz* locus, we drove a GFP reporter with a *CheB42a*/*llz* promoter, using the *UAS/GAL4* system [27]. Adults showed expression in male front legs (Fig. 2A), consistent with previous work showing enrichment in male appendages [6], [25]. Because the GFP signal appeared larger than we anticipated for a single cell, we co-expressed GFP with DsRed containing a nuclear localization signal [28]. Each GFP-positive spot included only one labeled nucleus, indicating expression in large, single cells rather than cell clusters (Fig. 2B). A careful morphological analysis suggested the labeled cells were probably not neurons because they lacked any detectable sensory cilia or neurites as were readily apparent in leg sensory neurons when the pan-neuronal *elav* promoter drove GFP expression (Fig. 2C). Furthermore, high-resolution confocal 3-D reconstruction (Movies S1 and S2) suggested the *CheB42a/llz*-expressing cells might be support cells. Support cells are thought to enwrap sensory neurons in the fly, although their role in sensory transduction is not well-understood [29]. These data are consistent with recent work suggesting that *CheB42a*-expressing cells are non-neuronal, and seem to be associated with cells expressing the putative pheromone receptor *Gr68a* [25]. They are also consistent with the recent discovery that ACD-1, another DEG/ENaC channel expressed in *C. elegans* and involved in sensing, is required in sheath, glia-like cells rather than neurons [30].

**Figure 3:**
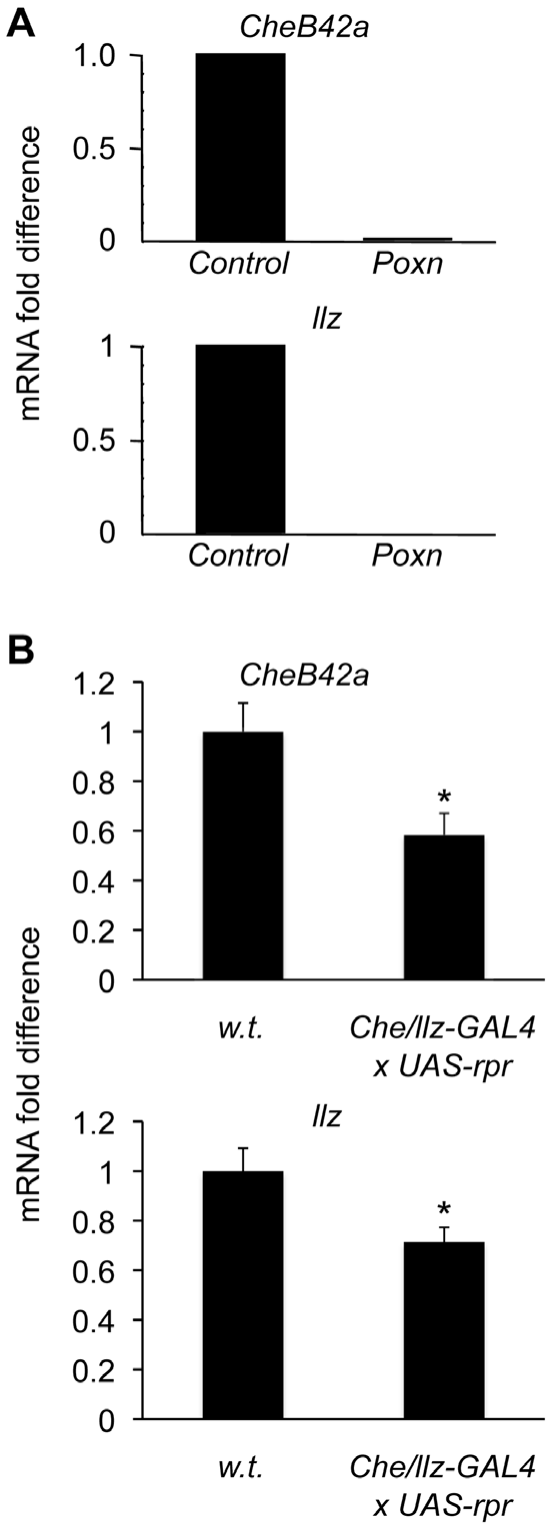
*CheB42a and llz* are expressed in the same chemosensory-related cells. **A.** The expression levels of both *CheB42a* and *llz* were reduced in homozygous *Poxn* mutants relative to heterozygote controls. Total RNA was extracted from male appendages and was analyzed by quantitative RT-PCR. Data are mRNA in *Poxn* relative to wild-type control. Expression levels were normalized to the housekeeping gene *rp49*. *y*-axis represents arbitrary mRNA fold-difference units, with control expression levels designated as 1 unit. **B.** Genetic ablation*CheB42a/llz*-expressing cells reduced expression of both transcripts. The *CheB42a promoter-GAL4* line was crossed to *UAS-rpr*, which induces cell-death [29]. Analysis as in panel A.

As an additional test of whether *CheB42a/llz* expressing cells are chemosensory in adults, we examined the expression of both genes in the *Poxn* mutant; this mutant converts most external chemosensory hairs into pure mechanosensory hairs [31]. The *Poxn* mutant reduced both *CheB42a* and *llz* transcripts (Fig. 3A). We also used the *CheB42a/llz* promoter and the *UAS/GAL4* system [27] to drive the apoptotic gene *rpr* [32], thereby genetically ablating cells expressing the locus. In these flies, levels of both transcripts fell (Fig. 3B), consistent with expression of both genes in the same cells. Involvement of a chemosensory system in non-neuronal cells bears similarity to vertebrate gustatory taste buds and the gustatory system in insects. Both systems have essential non-neuronal components that are involved in transmitting chemical signals [33].

**Figure 4:**
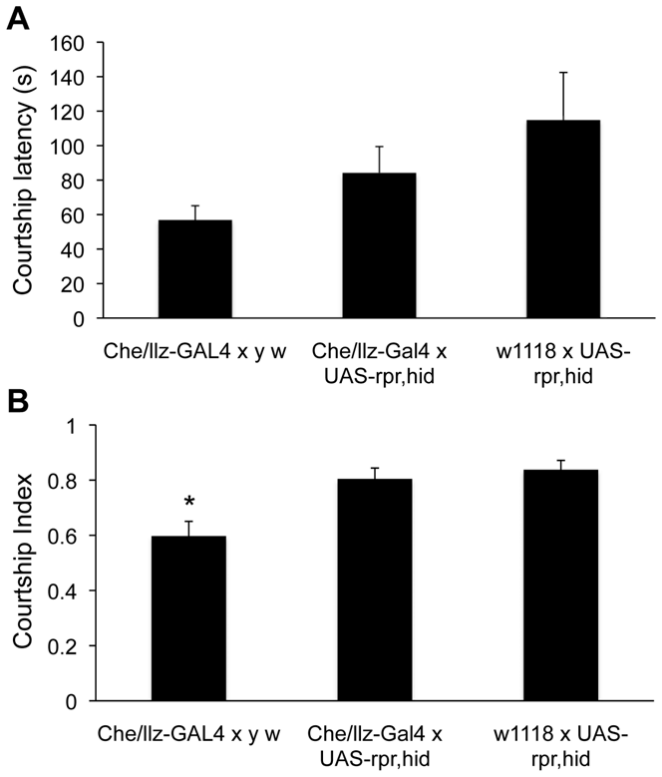
Genetic ablation of CheB42a/llz expressing cells does not affect male courtship behavior. **A.** Expression of the proapoptotic genes *hid* and *rpr* in *CheB42a/llz*-expressing cells using the UAS-GAL4 system did not affect male courtship latency (time from female introduction until the male shows any courtship related behaviors). Parental lines crossed to the reciprocal wild-type genetic background were used as controls. Although there were significant differences between the two parental controls, neither control line was statistically different from the focal cross (ANOVA). N  =  (*Che/llz*-GAL4 x *yw*, 33; *Che/llz*-GAL4 x UAS-*rpr, hid*, 52; *w^1118^* x UAS-*rpr, hid*, 27). **B.** Genetic ablation of *Che/llz*-expressing cells resulted in a mild effect on male courtship index (the proportion of time a male spends courting once courting started). Genotypes and sample sizes as in A. *, P<0.05.

Two previous studies suggested that *llz* and *CheB42a* might contribute to male courtship behavior, albeit with opposing effects on male-female interactions [7], [8]. Because our genetic ablation studies with transgenic expression of proapoptotic genes suggested that this technique reduced expression of the *CheB42a*/*llz* locus, we examined the effects of ablations on male courtship behavior. In contrast to previous reports [7], [8], our data suggested that ablating *CheB42a/llz*-expressing cells did not significantly change male courtship latency (Fig. 4A). However, we did observe a small effect on male courtship index, although the difference was significant only relative to one of the parental control crosses (Fig. 4B).

### CHEB42A Associates with LLZ

**Figure 5:**
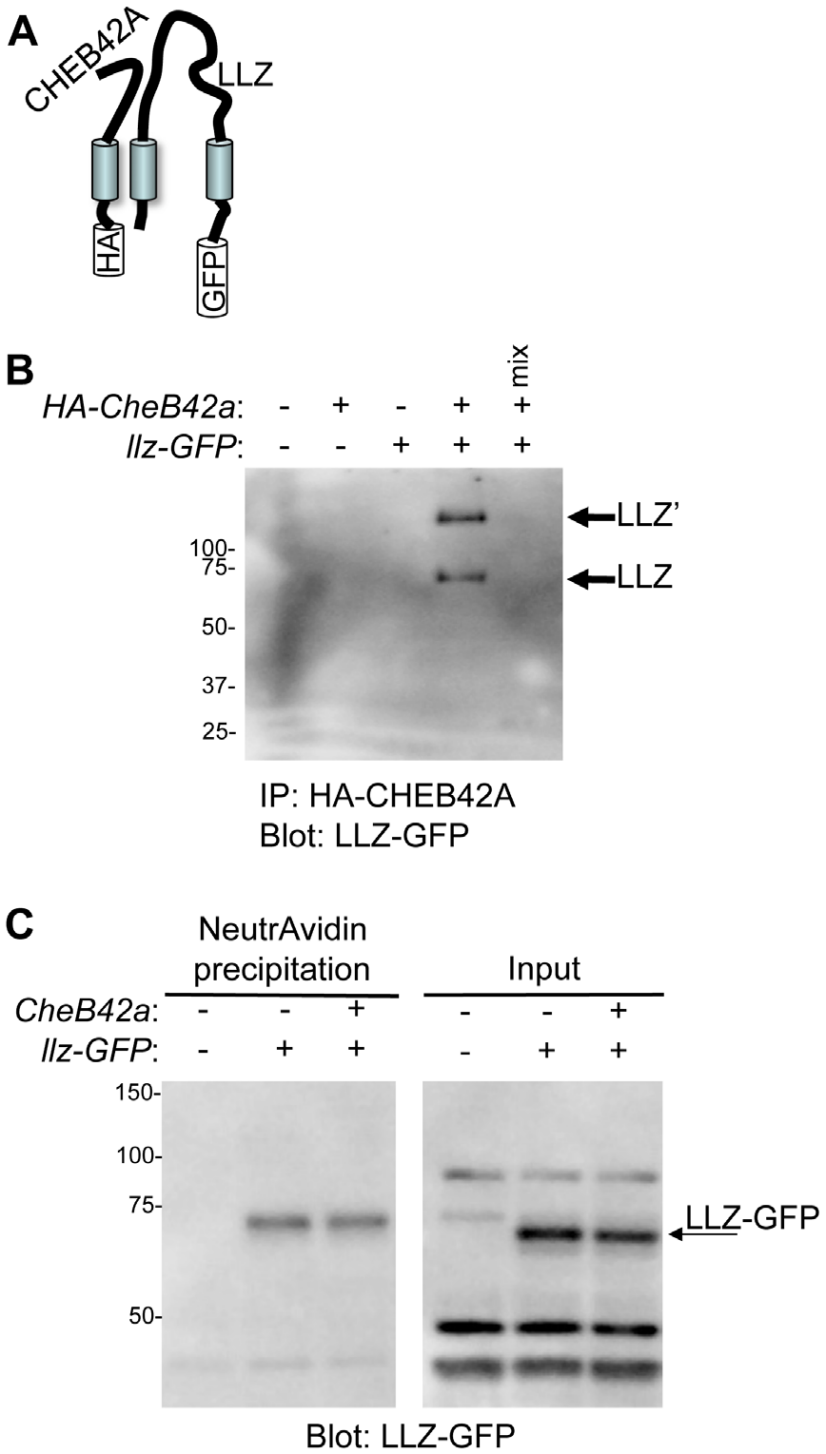
CHEB42A and LLZ can form a protein complex. **A.** Schematic of transfected proteins. **B.** Co-expression of tagged CHEB42A-HA and LLZ-GFP proteins. Immunoprecipitation with anti-HA antibody co-precipitated LLZ. “mix” indicates an experiment in which equal amounts of protein from the singly-transfected cells were mixed prior to immunoprecipitation as a control for non-specific interactions. Expression of *llz* produced two protein bands, the expected lower molecular mass band, plus a band of higher molecular mass. We do not know the identity of the more slowly migrating band; it might represent an LLZ-containing multiunit complex that is resistant to SDS denaturation or might represent post-translational modifications of LLZ [45]. We also attempted to detect HA-CHEB42A after immunoprecipitating LLZ-GFP, but were not successful. **C.** CHEB42A did not affect LLZ surface expression. COS7 cells were transfected with *llz* and either *GFP* or *CheB42a*. Surface expression level was estimated with biotinylation of surface proteins followed by neutravidin precipitation. To estimate total protein, protein input was directly blotted with anti-GFP antibody (right panel). We did not observe the larger LLZ' band in the surface expression study. We speculate that differences in sample processing protocols between the IP and surface expression studies could affect solubility and detection of larger protein complexes.

Since both *llz* and *CheB42a* are expressed in the same tissues and affect similar behavioral processes, we asked whether they are parts of the same protein complex. We tested whether CHEB42A could interact with LLZ by expressing tagged versions of both proteins in COS-7 cells. We then immunoprecipitated CHEB42A and found that LLZ co-precipitated (Fig. 5A,B). This result suggested that the two proteins could form a complex.

To test whether LLZ alone or together with CHEB42a forms an ion channel, we expressed them alone and together in *Xenopus* oocytes and in CHO cells and measured current with two-electrode voltage clamp or patch-clamp, respectively. These experiments failed to reveal *llz*-dependent currents (not shown). Mutations in the residue preceding the second transmembrane domain of some DEG/ENaC subunit can generate a constitutively open channel (a “DEG” mutation) [34]. Interestingly, LLZ contains a methionine at this position (M409), which would predict that LLZ might have a “natural” DEG mutation and hence should behave as a constitutively open channel. Yet, adding amiloride (up to 100 µM), which inhibits some DEG/ENaC channels, did not reduce current. Moreover, extracellular addition of acid, hyper- and hypotonic solutions, FMRFamide, or proteases did not generate currents in cells expressing *llz* alone or with *CheB42a*. Because *llz* and *CheB42a* were both speculated to function in pheromone sensing pathways, we also tested whether LLZ might be gated directly by virgin female pheromonal hexane extracts, but we were not able to observe any *llz*-dependent currents. These electrophysiological experiments failed to induce current irrespective of expression in oocytes or CHO cells and regardless of whether *llz* was expressed alone or with *CheB42a*.

We considered that these experiments may have been negative because we do not know the appropriate ligand to activate LLZ and/or CHEB42a, or because LLZ must heteromultimerize with another as yet unknown DEG/ENaC subunit to generate current. Alternatively, it is possible that our expression constructs did not express enough *llz* transcripts to detect currents in oocytes. However, the same constructs expressed well in mammalian cells, but likewise did not elicit *llz*-dependent currents there. In addition, the same vectors were used to express ASIC1a in oocytes. Together, these data suggested that lack of *llz* expression is an unlikely explanation for the absence of *llz*-dependent currents in oocytes.

Because LLZ and *CheB42a* can associate, we asked if CHEB42A alters the presence of LLZ in the cell membrane. To test this possibility we biotinylated cell membrane proteins and precipitated with neutravidin. LLZ was precipitated, indicating its presence on the cell surface. However, co-expressing *CheB42a* did not alter the amount of biotinylated and precipitated LLZ (Fig. 5C). Thus, despite the interaction of the two proteins, CHEB42A did not induce LLZ channel activation or alter the relative amount of protein on the cells surface.

### CHEB42A Modifies the Current of Mammalian ASIC1a Channels

**Figure 6:**
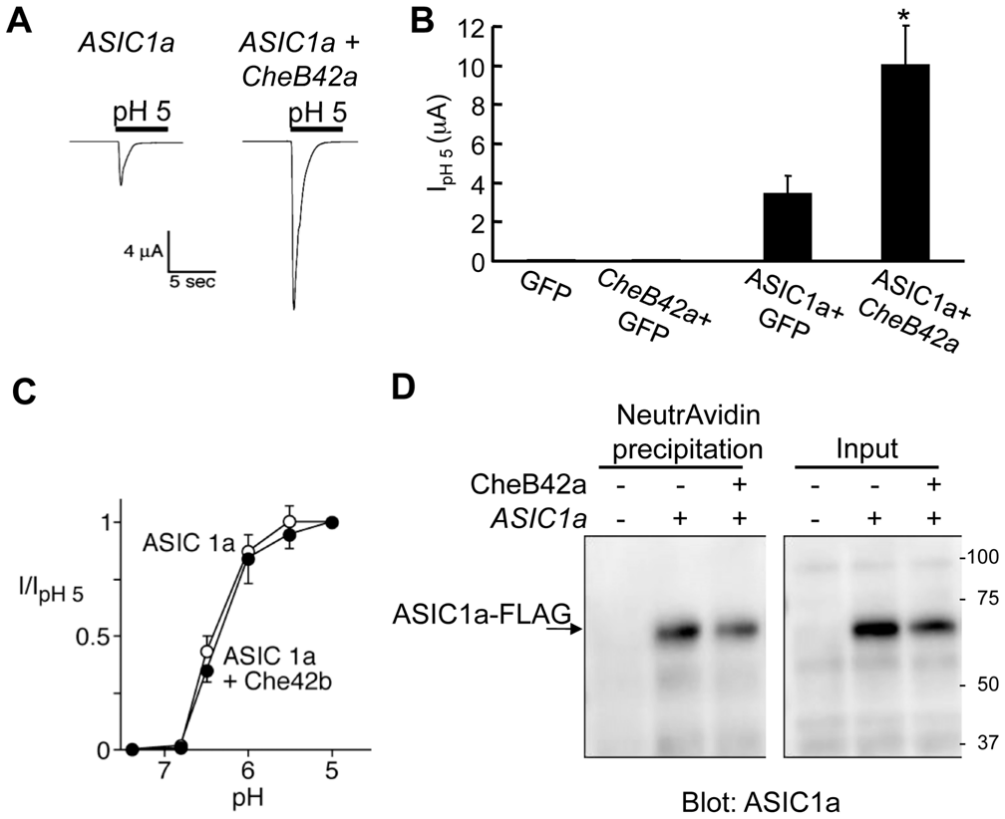
CHEB42A increases current from the mammalian DEG/ENaC protein ASIC1a. **A–C.** Effect of CHEB42A on H^+^-gated ASIC1a currents. *Xenopus* oocytes were injected with *ASIC1a*, *GFP* or *CheB42a* cDNAs alone or in combinations indicated. Bathing solution pH was reduced to 5 during time indicated. **A.** example of H^+^-gated currents from the ASIC1a channel. **B.** CHEB42A increases ASIC1a H^+^-gated currents. Asterisk indicates P<0.01 (two-tail *t*-test). Number of oocytes studied: 9 *GFP*, 9 *CheB42a* and *GFP*, 20 *ASIC1a* and *GFP*, and 27 *ASIC1a* and *CheB42a*. Data are mean ± SEM. **C.** CHEB42A did not affect the pH dependence of ASIC1a current. Closed symbols indicate ASIC1a and CHEB42A (n = 15) and open symbols indicate ASICla + GFP (n = 12). **D.** CHEB42A did not affect ASIC1a surface expression. Experiment was done as in Fig. 5B.

While we do not know the ligand for LLZ, we do know the ligand for some other DEG/ENaC channels. For example, extracellular protons activate another Deg/ENaC channel, the acid-sensing ion channel-1a (ASIC1a) [35]. Therefore, we asked whether CHEB42A might affect the activity of ASIC1a. As previously described [36], [37], ASIC1a produced acid-evoked currents when expressed in *Xenopus* oocytes (Fig. 6A,B). However, when we co-expressed CHEB42A with ASIC1a, acid-evoked currents increased three-fold. CHEB42a did not alter the pH-sensitivity of ASIC1a current (Fig. 6C). As we had observed with LLZ, CHEB42a did not increase the amount of ASIC1a on the cell surface (Fig. 6D). These results are similar to the report that the *C. elegans* DEG/ENaC accessory subunit MEC-6 increases current of the Deg/ENaC channel MEC-4/MEC-10 without altering the cell surface expression of either subunit [17], [21].

### *CheB42a* Encodes a Secreted Peptide

**Figure 7:**
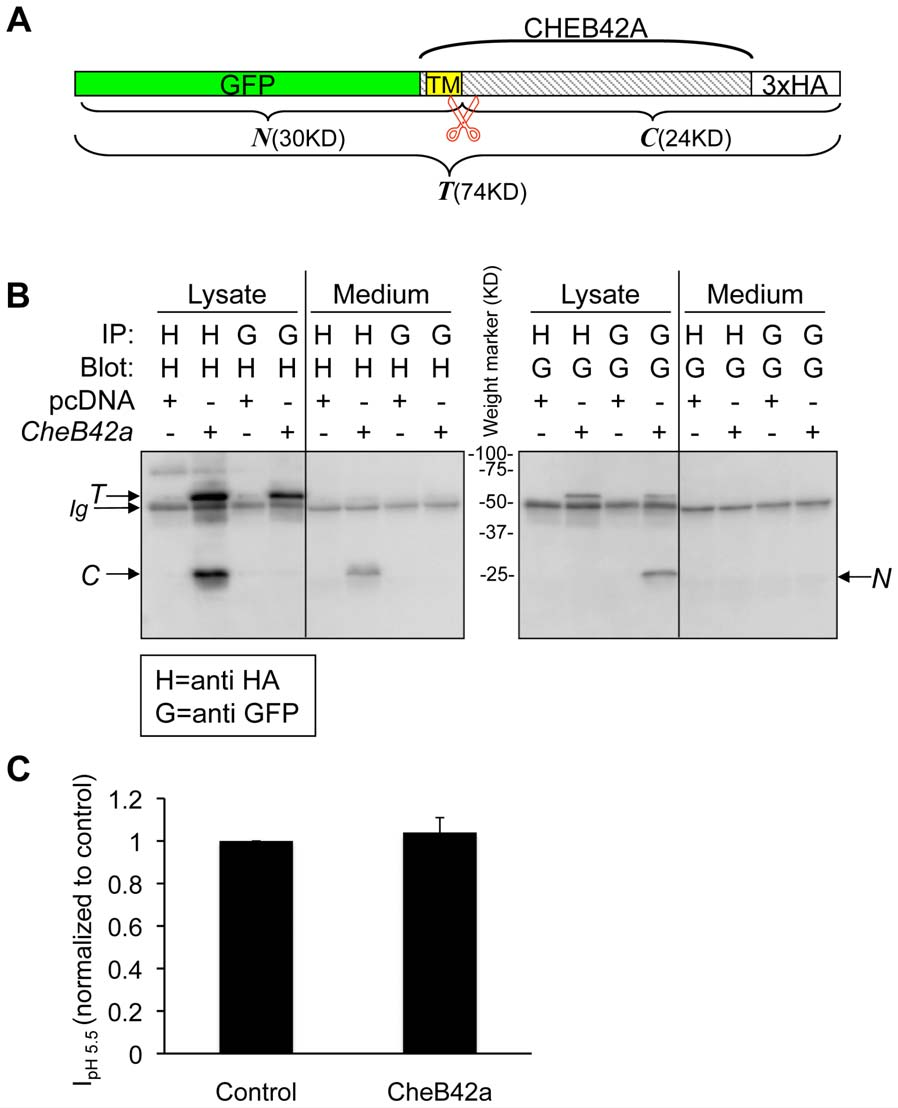
CHEB42A produces a secreted peptide. **A.** Schematic of the construct used to study CHEB42A. *CheB42a* cDNA was tagged with GFP at the 5′ end and 3xHA tags at the 3′ end and was cloned into the mammalian expression vector *pcDNA3.1* (Invitrogen). TM, transmembrane peptide as predicted by the SignalP 3.0 Server (http://www.cbs.dtu.dk/services/SignalP/). predicted transmembrane domain. 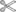 Predicted signal peptide cleavage site. *T*, *N*, and *C* represent the predicted full length and cleaved products. **B.** CHEB42A is a secreted protein. Cells were transfected with tagged cDNA (*CheB42a*) or *pcDNA3.1* as a control. Proteins from cell lysates or cell media were immunoprecipitated (IP) with either anti-GFP (G) or anti-HA (H) antibodies, and then blotted for either tag. *T*, *N*, and *C* indicate the predicted peptides from Fig. 7A. **C.** Effects of *CheB42a*-conditioned media on ASIC1a currents. ASIC1a was expressed in *Xenopus* oocytes as in Fig. 6. Four ASIC1a-expressing oocytes were tested for pH-dependent currents with and without conditioned media from *CheB42a*-expressing cells. The same oocytes (N = 4) were stimulated with unconditioned medium (pH 5.5), followed by stimulation with *CheB42a*-conditioned medium. There was no obvious effect of the conditioned media on pH-activation of ASIC1a channels. Currents were normalized in the conditioned state relative to the control media. All oocytes showed significant ASIC1a-dependent currents in response to pH 5.5, which does not elicit any currents in non-injected oocytes.

Sequence analysis of CHEB42A with the SignalIP algorithm suggested that the transmembrane domain might function as a signal peptide [38], raising the possibility that the extracellular domain of the protein might be secreted. To test the hypothesis that CHEB42A might generate a secreted peptide, we constructed a *CheB42a* cDNA that carried a GFP at its predicted intracellular N-terminus and a 3xHA tag at its predicted extracellular C-terminus (Fig. 7A).

We expressed these constructs and tested for the tagged protein fragments in both the cellular fraction and the cell culture medium. The cell lysate contained the full-length protein plus N- and C-terminal fragments (Fig. 7B). In addition, we detected the C-terminal extracellular portion of CHEB42A in medium covering the cells. In contrast, the N-terminal fragment was detected only in the cell lysate. These results suggest that the extracellular domain of CHEB42A can be secreted. These data also indicate that the cleavage of CHEB42A occurs intracellularly since we were able to detect both N- and C-terminal fragments in cell lysates.

Because *CheB42a* can generate a secreted protein, we asked whether the secreted form might alter ASIC1a-dependent currents. We examined ASIC1a activation in response to lowered extracellular pH in the presence or absence of conditioned media from *CheB42a*-expressing cells. The conditioned media had no effect on pH-dependent currents (Fig. 7C), suggesting that the modulatory effect of CHEB42A was not mediated by the secreted peptide, but rather required physical interaction with the full-length, membrane bound protein.

## Discussion

We previously identified operon-like cotranscription of the *CheB42a* and *llz* loci [4]. In addition, earlier studies suggested that *CheB42a* and *llz* are expressed in similar tissues and they might contribute to male courtship behavior [5], [6], [25], [39]. Here we show that as with other operons, the two gene products biochemically interact and may be functionally related.

Our data suggest that CHEB42A may function as an accessory subunit for DEG/ENaC channels. Although we were not able to elicit current from LLZ, we found that CHEB42A increased the current amplitude from another DEG/ENaC channel, ASIC1a, for which the ligand is known [14], [40]. In addition, CHEB42A associated with both LLZ and ASIC1a. How CHEB42a influences ASIC1a current remains uncertain, but our data suggest that CHEB42A does not increase the amount of LLZ or ASIC1a at the cell surface, nor did it change the pH-sensitivity of ASIC1a. Furthermore, although CHEB42A can be secreted, the modulatory effects on DEG/ENaC currents were not affected by the secreted peptide alone, suggesting that CHEB42A may have multiple, distinct physiological roles. These results are similar to the finding that the *C. elegans* MEC-6 protein increases current amplitude through the DEG/ENaC channel MEC-4/MEC-10 without altering its surface-expression, and all three proteins are required for mechanosensation [21]. It is also interesting that both CHEB42A and MEC-6 have extracellular domains with sequence similarities to enzymes; CHEB42A shares limited sequence similarity to aryl sulfotransferases of the SULT1A subfamily, and MEC-6 shows similarity to paraoxonases [21]. However, neither CHEB42a nor MEC-6 is known to have enzymatic activity. The fly genome contains 30 DEG/ENaC genes and 12 *CheB* genes. Our data raise the interesting possibility that CHEB42A or other members of the *CheB* family could regulate other channels. Perhaps combinatorial expression of members of the two families is a mechanism to increase receptor diversity and function.

Finding that CHEB42A is cleaved and the extracellular domain is released into the medium suggests that CHEB42A might have a function in addition to that of an accessory subunit. In this regard, CHEB42A is similar to several odorant-binding proteins, which are also expressed in and secreted from non-neuronal chemosensory accessory cells [41]. We speculate that it might interact with a chemical ligand produced by female flies and passed by physical contact to male front legs, where it could influence LLZ channel function, or possibly other sensory receptors. Interestingly, a recent study suggested that *CheB* genes might encode proteins that are homologs of the mammalian Tay-Sachs GM2-activator protein, a lysosomal soluble co-factor involved in the degradation of the ganglioside GM2 [19]. Our data did not support this hypothesis since we find that CHEB42A is a secreted rather than a soluble lysosomal protein. Yet, it is possible that some of the effects of CHEB42A on Deg/ENaC functions are to enzymatically modify external ligands, which can modulate channel function.

How the *CheB42a*/*llz* locus influences courtship behavior remains uncertain. There are several considerations. First, both genes were expressed in the forelegs of males, consistent with a role in male courtship behavior. Second, previous studies suggested that both genes can influence male courtship behavior, but unexpectedly, *llz* mutations delayed and *CheB42a* mutations enhanced courtship behavior [7], [8]. Third, rather than neurons, the *CheB42a/llz* locus was expressed in support cells enwrapping sensory neurons [29]. This localization is interesting given the recent surprising discovery that a *C. elegans* DEG/ENaC channel is required in glia rather than neurons to influence sensory function [30]. Fourth, our data suggested that genetic ablation of *CheB42a/llz*-expressing cells had little effect on male courtship behavior. Thus, we speculate that *CheB42a* and *llz* modulate chemosensory neuron function from a location in support cells. Thus, the support cells may not be required for the chemosensation, but through *CheB42a* and *llz*, they can modify the function of the neurons they enwrap.

Our current results further support the conclusion that the *CheB42a*/llz locus has operon-like transcription. In addition, a recent report suggested that another chemosensory-related locus in the fly genome is transcribed as a polycistronic mRNA [42]; the *Gr64a-f* locus encodes several sugar receptors. Finding that sensory-related loci are co-transcribed suggests an evolutionary solution for finely controlling the spatial, temporal and quantitative aspects of chemosensation. Tight control on the expression of membrane-bound complexes is probably essential in many eukaryotic systems. Hence, these results also raise the intriguing possibility that operon-like transcription may be more common in eukaryotes than has previously been appreciated, including organisms outside the *Drosophila* lineage. We speculate that identification of other co-transcribed genes may reveal novel protein interactions and pathways.

## Materials and Methods

### *Drosophila* Stocks and Cultures

Fly stocks and crosses were maintained according to standard *Drosophila* culture procedures and were housed in a 25 °C incubator under 12∶12 light:dark cycle. A *CheB42a promoter-GAL4* line was a gift from C.W. Pikielny. We generated additional *CheB42a*/*llz promoter-GAL4* lines by PCR amplifying a ∼2.5 kb fragment which included upstream sequences and the first intron of *CheB42a* subcloned into a *pPTGAL4* vector (a gift from D. Eberl). The *elav*-*GAL4*, *UAS-EGFP*, *UAS-rpr*, and *UAS-stinger* (nuclear DsRed) were obtained from the Bloomington Fly Stock Center (IN). The *UAS-hid, UAS-rpr* line was from P. Taghert. The *Poxn* ^Δm^ flies were a gift from M. Noll.

### RNA Analysis

Flies were separated by sex under CO_2_ and kept at −80°C until processing. To separate body parts, microcentrifuge tubes with flies were dipped in liquid nitrogen and then separated by repeated vortexing. Total RNA was extracted with the RNeasy mini kit (Qiagen) or TRizol reagent (Invitrogen) according to manufacturer instructions. Real-time quantitative RT-PCR assays were performed on an “ABI7500 fast” with pre-designed real-time FAM-labeled probe-based assays from ABI according to manufacturer instructions. For analysis, expression levels of the housekeeping gene *rp49* were used as an RNA loading control. Data were transformed according to the ΔΔCt method and are represented as relative values [43]. Fold difference was calculated relative to lowest-expressing sample, which represented 1 arbitrary unit of expression.

ABI assays used were: *rp49*, Dm02151827_g1; *CheB42a*, Dm01794214_g1; *llz*, Dm01794217_g1.

### Co-Immunoprecipitation and Cell Surface Expression of Protein

COS-7 or HEK293T cells were electroporated with 12 µg of either control *pEGFPN1*, *pcDNA3.1*[*CheB42a-HA*], *pEGFPN1[llz*], or both *pcDNA*3.1[*CheB42a*-HA] and *pEGFP*[*llz*] (6 µg each), and were then maintained in standard medium in a 37°C incubator for 48 h. For co-immunoprecipitation, cells were lysed in RIPA buffer containing 1% NP-40 (Pierce). Extracted protein was first incubated with either anti-HA or anti-GFP antibodies for 3 h followed by 1 h incubation with protein A sepharose (Pierce). Bound protein was released from pelleted protein A by direct incubation with 2% SDS loading buffer and analyzed according to standard western blotting techniques. Total cell protein was measured using the BCA assay (Pierce). Analysis of expression of proteins on the cell surface was done with the EZ-Link-NHS-Biotin reagent according to manufacturer instructions (Pierce). Total protein for surface expression normalization was measured by western blotting of total protein input.

To measure CHEB42A secretion, the full-length *CheB42a* cDNA minus the STOP codon was amplified using gene specific primers. The reverse primer included the sequence for 3xHA tag such that the expressed protein will be tagged with a 3xHA on its C' terminus. The PCR product was then cloned into a pEGFP-C vector which resulted in a final expressed protein that has a GFP tag on its N' terminus (intracellular) and 3xHA tag on it C' terminus (extracellular). The construct was transfected into HEK293-T cells and proteins were extracted according to standard protocols as described above. To harvest secreted protein in the conditioned media, medium from cells expressing the pEGFP-CheB42a-3xHA construct were concentrated using Amicon Ultra-4 Centrifugal Filters according to manufacturer instructions.

### Electrophysiology

To test the effect of CHEB42A on LLZ and ASIC1a, we injected nuclei of albino *Xenopus laevis* oocytes with 20 nl of water containing cDNAs (in *pMT3-Swick* [44]) encoding ASIC1a (0.02 mg/ml) or LLZ (0.02 mg/ml) and CHEB42A or GFP (0.04 mg/ml). Following incubation for 16–24 hrs in modified Barth's solution at 18°C, we measured whole-cell current by two-electrode voltage-clamp at −60 mV. Oocytes were bathed in pH = 7.4 NaCl Ringer (116 mM NaCl, 2 mM KCl, 0.4 mM CaCl_2_, 1 mM MgCl_2_, 5 mM Hepes). Maximal H^+^-activated current was measured by perfusing cells with pH = 5 NaCl Ringer. We also expressed *llz* alone and with *CheB42a* in Chinese hamster ovary (CHO) cells and measured current with the whole-cell, patch-clamp technique as we have previously described [37].

## Supporting Information

Movie S1 A 3D movie of a confocal z-stack reconstruction of a single *Che/llz* positive cell in a male foreleg. Flies expressed simultaneously a nuclear localized DsRed and cytoplasmic GFP proteins.
(1.62 MB AVI)

Movie S2 A 3D movie of a confocal z-stack reconstruction of a single *Che/llz* positive cell in a male foreleg. Flies expressed simultaneously a nuclear localized DsRed and cytoplasmic GFP proteins. Images in Movies S1 and S2 were recorded from two independent cells in a single male.
(0.69 MB AVI)
